# Characterization of Pharmaceutical Tablets Using UV Hyperspectral Imaging as a Rapid In-Line Analysis Tool

**DOI:** 10.3390/s21134436

**Published:** 2021-06-28

**Authors:** Mohammad Al Ktash, Mona Stefanakis, Barbara Boldrini, Edwin Ostertag, Marc Brecht

**Affiliations:** 1 Process Analysis and Technology PA & T, Reutlingen University, Alteburgstraße 150, 72762 Reutlingen, Germany; Mohammad.Alktash@reutlingen-university.de (M.A.K.); Mona.Stefanakis@reutlingen-university.de (M.S.); Barbara.Boldrini@reutlingen-university.de (B.B.); Edwin.Ostertag@reutlingen-university.de (E.O.); 2 Institute of Physical and Theoretical Chemistry, Eberhard Karls University Tübingen, Auf der Morgenstelle 182, 72076 Tübingen, Germany

**Keywords:** hyperspectral imaging, pushbroom, UV spectroscopy, principle component analysis, active pharmaceutical ingredient API, process analytical technology PAT

## Abstract

A laboratory prototype for hyperspectral imaging in ultra-violet (UV) region from 225 to 400 nm was developed and used to rapidly characterize active pharmaceutical ingredients (API) in tablets. The APIs are ibuprofen (IBU), acetylsalicylic acid (ASA) and paracetamol (PAR). Two sample sets were used for a comparison purpose. Sample set one comprises tablets of 100% API and sample set two consists of commercially available painkiller tablets. Reference measurements were performed on the pure APIs in liquid solutions (transmission) and in solid phase (reflection) using a commercial UV spectrometer. The spectroscopic part of the prototype is based on a pushbroom imager that contains a spectrograph and charge-coupled device (CCD) camera. The tablets were scanned on a conveyor belt that is positioned inside a tunnel made of polytetrafluoroethylene (PTFE) in order to increase the homogeneity of illumination at the sample position. Principal component analysis (PCA) was used to differentiate the hyperspectral data of the drug samples. The first two PCs are sufficient to completely separate all samples. The rugged design of the prototype opens new possibilities for further development of this technique towards real large-scale application.

## 1. Introduction

A large number of remote sensing applications have been developed over the last decade [1]. This also led to establish non-destructive imaging systems that are able to quickly identify quality problems within the scanned area [2,3]. Spectral imaging involves both spectral and spatial information of any particular sample or region within an area of interest, thus each pixel represents spectral and spatial information. Imaging systems can be realized in the modes of hyperspectral and multispectral imaging. The difference between these modes is the number and width of the recorded spectral bands. In multispectral imaging 3–10 bands are used [4]. In hyperspectral imaging hundreds or thousands of correspondingly more narrow bands are employed [5,6,7,8]. Therefore, hyperspectral imaging is also known as imaging spectroscopy, a technique that combines conventional imaging with spectroscopy [6]. Hyperspectral imaging setups produce a 3D data matrix often referred to as hypercube. Two of the dimensions are reserved for the spatial information (*x*, *y* coordinate) while the third dimension represents the spectroscopic information (*λ* coordinate) [6,9,10].

Hyperspectral imaging is not restricted to the visible range, nowadays high performance systems are also available for the near infrared range (NIR) [6,11]. Hyperspectral imaging is a rapid and non-destructive method which analyzes samples without changing their physical shape. This robust technique in combination with real-time chemometric analysis can be easily integrated into an industrial production environment. This enabled chemical sensing systems for very different applications in the fields of food quality monitoring, textile classification, agriculture, detection target of military, astronomy, life science, medicine and pharmaceutical drugs [2,3,12,13,14,15]. Traditional methods such as UV-Vis spectroscopy, high performance liquid chromatography (HPLC) or mass spectrometry (MS) are, in contrast, time consuming, expensive and require sample preparation and destruction [2,6]. Very recently, Tschannerl et al. reported an interesting application of hyperspectral imaging in UV range. They were able to precisely discriminate between phenolic flavor concentrations in melted barley by using hyperspectral imaging in UV and NIR regions [6].

In 2004, the food and drug administration (FDA) in the US started the Process Analytical Technology (PAT) initiative to control manufacturing processes [2]. Hyperspectral imaging is an attractive PAT tool for the quality assurance of final products. Hyperspectral imaging, as expected, will be increasingly used as a PAT tool in the industry; it has been already applied in the industrial manufacturing of pharmaceutical drugs and quality control of pharmaceutical products [16,17]. Most drugs appear colorless to the eye, meaning that they do not absorb light in the visible region but they may absorb in the UV region according to the chemical structure [18]. Such drugs as ibuprofen (IBU), acetylsalicylic acid (ASA) and paracetamol (PAR) show certain absorbance in UV region [19]. Up to now, a variety of drug studies in the UV-Vis region have been performed; Saeed et al. investigated the active pharmaceutical ingredients paracetamol, aspirin, ibuprofen, codeine and caffeine in different formulations by UV-Vis spectroscopy [20]. Rote et al. developed a method to simultaneously quantify paracetamol and nabumetone by area under curve in bulk and tablet dosage form [21].

Hyperspectral imaging setups acquire thousands of spectra in short time resulting in a massive amount of data. Therefore, techniques for data evaluation like the principle component analysis (PCA) methods are required. PCA is one of the most common statistical methods. This technique is used for data evaluation/reduction but simultaneously minimizing information loss in spectroscopy [22,23]. In addition, it is capable of visualizing common features in the data set to detect possible groups and their heterogeneity within samples [24]. PCA combined with hyperspectral imaging data can highlight the relative distributions of different components in mixtures and reveal the spectral features in the spectroscopic data [9,25].

The aim of this study is to develop a hyperspectral imaging system in the UV wavelength range for the in-line characterization of pharmaceutical tablets. The results show that hyperspectral imaging in the UV range is a suitable technique for in-line measurements with the aim of a real-time classification at short time and low costs.

## 2. Materials and Methods

### 2.1. Samples

**Figure 1:**
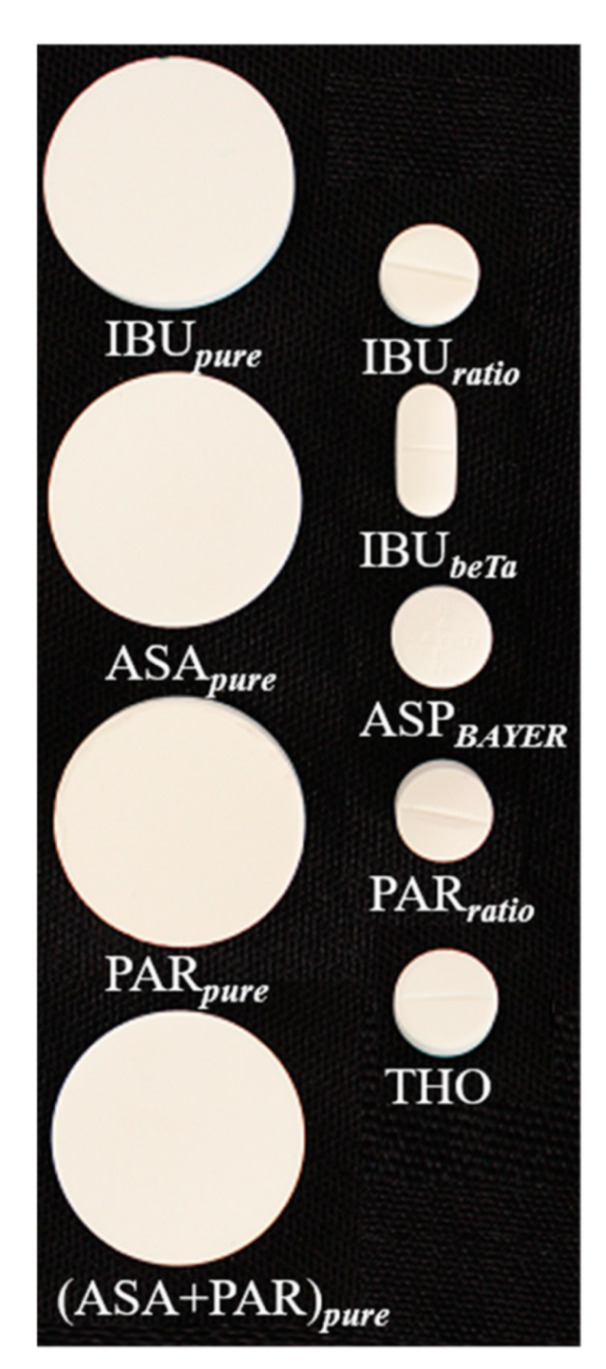
Drug samples. Left column: Reference API samples, right column: painkiller tablets.

Two groups of samples were analyzed. Figure 1 shows all tablets used. Further information is listed in Table 1. In the following, these samples are referred to as IBU*_pure_*, ASA*_pure_*, PAR*_pure_*, IBU*_ratio_*, IBU*_beTa_*, ASP*_BAYER_*, PAR*_ratio_* and THO. For hyperspectral imaging measurements, the coating of the commercially available painkiller tablets was removed by sandpaper manually (grain size 320, Emil Lux GmbH & Co. KG, Wermelskirchen, Germany). For each removal step a new stripe was brushed over it twice. The painkiller tablets were measured at different depths. A layer of approximately 500 μm ± 50 μm thickness was removed from the samples after each measurement. Three samples of each type were collected (painkiller samples) and created (pure API samples) for the study.

In total, 4 g of ASA*_pure_* and IBU*_pure_* were pressed at 10 tons for 2 min by a hydraulic press (PerkinElmer, Inc., Waltham, MA, USA) into the depicted disc shape. Then, 4 g PAR*_pure_* powder were dried in a vacuum oven (VACUTHERM, Thermo Scientific, Waltham, MA, USA) for 1 h at 120 °C, and pressed at 10 tons for 20 min (see Table 1). A mixture of 2 g ASA*_pure_* and 2 g PAR*_pure_* was prepared by using a SpeedMixer^TM^ (DAC 150.1 CM41, Hauschild GmbH & Co KG, Hamm, Germany), and pressed at 10 tons for 2 min.

### 2.2. API’s in Solution

A solution of ASA*_pure_* (100 µg mL^−1^) was prepared by dissolving 50 mg ASA*_pure_* in 500 mL of 0.1 M HCl:methanol (1:1) in 500 mL volumetric flask with strong shaking.

For PAR*_pure_* and IBU*_pure_* solutions, 10 mg of each API were dissolved in 15 mL methanol by shaking. Then, 85 mL water was added to adjust the volume up to 100 mL (resulting to 100 ppm). From that, 5 mL were taken, and volume was adjusted up to 50 mL with diluent [20].

### 2.3. UV Spectroscopy

Total (specular and diffuse) reflectance spectra of all samples (pure API and painkiller tablets) were recorded in the range of 200–380 nm using a commercial UV spectrometer (Lambda 1050+, PerkinElmer, Inc., Waltham, MA, USA). Both sides of the pure API samples were measured. The UV-Vis/NIR spectrometer was equipped with a 150 mm Spectralon^®^ integrating sphere to acquire data in reflection mode with an R6872-Photomultiplier (PMT). A deuterium lamp was used as light source in the spectrometer. The samples were placed at the reflectance port of the integrating sphere with a diffused scattering Spectralon^®^ disk placed behind the samples. The port measuring area is approximately 4.9 cm².

Absorbance spectra were measured using the aforementioned spectrometer in the range of 200–320 nm connected to the transmittance accessory. The liquid samples were measured at 2 nm spectral resolution. A 1 mm quartz SUPRASIL^®^ cuvette (106-1-K-40, Hellma, Müllheim, Germany) was used for measuring the API’s in solution.

Fluorescence excitation spectra were recorded by using a commercial setup (Fluorolog–3, HORIBA, Kyoto, Japan). The system includes a double grating monochromator in the excitation (*λ_Ex_* = 270 nm) and emission (*λ_Em_* = 280 nm–380 nm) paths in an “L” configuration. A 10 mm quartz SUPRASIL^®^ cuvette (111-10-K-40, Hellma, Müllheim, Germany) was used for measuring the samples.

### 2.4. UV Hyperspectral Imaging

**Figure 2:**
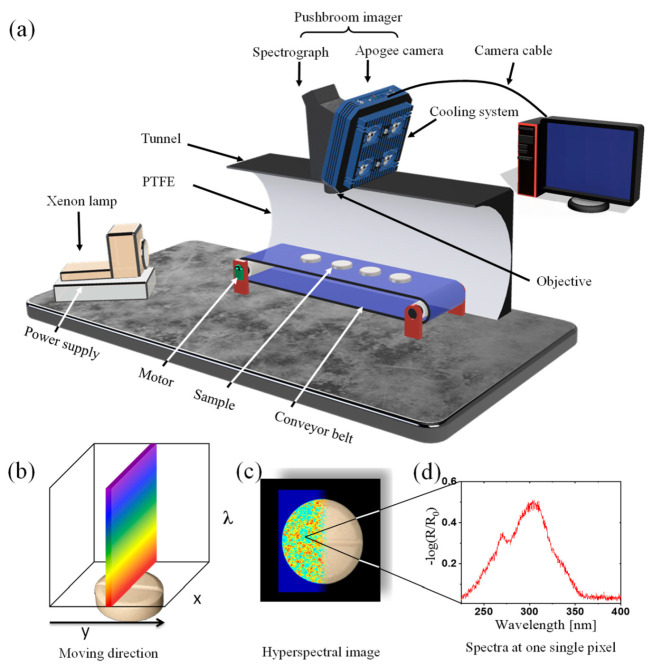
(**a**) Setup of a hyperspectral imaging system based on the pushbroom concept (the tunnel in the scheme was cut to show the inside). (**b**) Pushbroom Imager scanning principle. (**c**) Hyperspectral image generated immediately from the scanning of a sample. (**d**) UV spectrum for one single pixel extracted from the image given in (**c**).

Figure 2a shows a scheme of the hyperspectral imaging setup. The setup is based on a spectrograph (RS 50-1938, inno-spec GmbH, Nürnberg, Germany) connected to a CCD camera (Apogee Alta F47: Compact, inno-spec GmbH, Nürnberg, Germany) with 300 ms integration time. The samples were placed on a conveyor belt moving with speed 0.3 cm/s, which was positioned completely in a tunnel made of PTFE. The purpose of the tunnel design is to have an easily accessible system, which also ensures diffuse illumination of the samples and maintains a reasonable illumination strength and homogeneity. This minimizes an influence of the sample shape and roughness on the spectra. The illumination is provided by a Xenon lamp (XBO, 14 V, 75 W, OSRAM, München, Germany). Figure 2b–d illustrates the principle and workflow of the data acquisition. The continuous line by line collection of spectral information enables a lateral (*x*, *y*) 2D image as shown in Figure 2c, whereas each pixel contains a further spectroscopic dimension (*λ*) as shown in Figure 2d. Thus, a 3D data matrix (hypercube) is recorded.

### 2.5. Data Collection and Preprocessing

**Figure 3:**
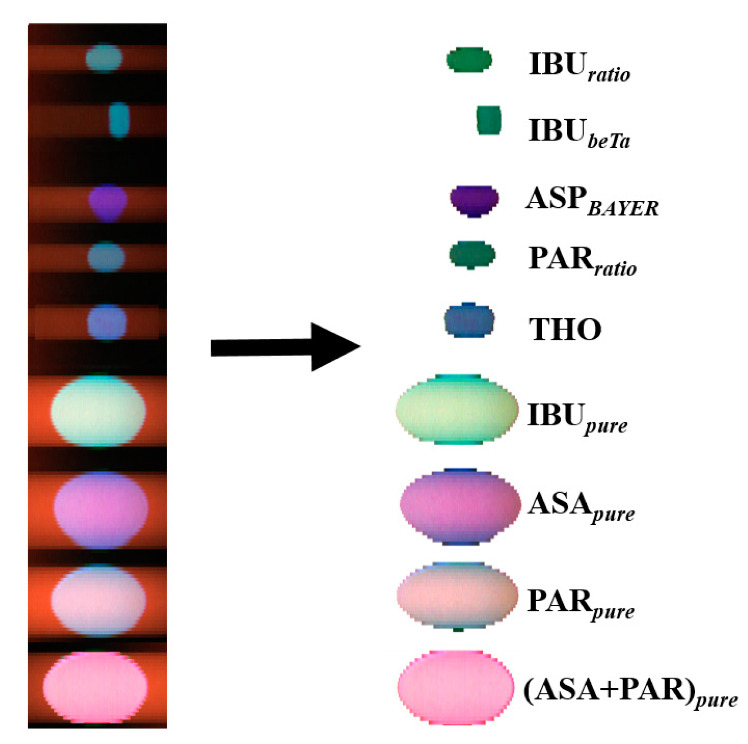
Hyperspectral raw image of nine drug samples on the left. Images after subtraction of the background on the right.

Figure 3 shows the original images of the drug samples before and after background subtraction. The UV hyperspectral images are captured by moving the drug samples at constant speed. For the collection of UV hyperspectral imaging data set one sample of each type was chosen randomly.

A distinction between the respective spectral characteristics was made first to differentiate signal and background. For this purpose, the regions assigned to the drug samples were manually selected to eliminate the signals from background. The remaining hypercube was used as input for the subsequent PCA classification.

### 2.6. Data Handling and Software

The UV spectra were recorded with the Lambda 1050 UV WinLab software from PerkinElmer. The UV hyperspectral imaging data were analyzed by the SI-Cap-GB version V3.3.x.0 software (inno-spec GmbH, Nürnberg, Germany). Hyperspectral data matrices were analyzed by Prediktera Evince version 2.7.11. PLS_Toolbox (PLS Toolbox 8.5.1, Eigenvector Research, Inc., Wenatchee, WA, USA) and MATLAB (MATLAB 9.2.0, Mathworks, MA, USA) were used for the data processing and analysis. An initial baseline correction was followed by a Savitzky-Golay 1st derivative (15 points, 2nd polynomial order). PCA models were calculated with cross validation (venetian blinds, 10 splits, 1 sample per split) and mean centering. A PCA combined with a quadratic discriminant analysis (QDA, 2 PCs) was calculated by using the software Unscrambler X 10.5 (Camo Analytics AS, Oslo, Norway) including the same spectral preprocessing.

Lighting conditions may vary between the samples and even within the samples across the scan line. A regular way to reduce this effect is to convert measured raw spectra to reflectance spectra by the following formula [6,11]:

(1) $$\mathrm{Reflectance}=\frac{R}{R_{0}}=\frac{I_{\mathrm{sample}}\;-\;I_{\mathrm{dark}}}{I_{\mathrm{reference}}\;-\;I_{\mathrm{dark}}}$$

where R and R_0_ represent the reflected intensity by the sample and a specific reference material with high reflectance capability. I_sample_ is the intensity of the original image data, I_dark_ is the intensity of the dark current image data and I_reference_ is the intensity of the white reflectance image [3]. For a better comparison of the reflectance spectra to the extinction spectra in solution (absorbance) the negative decadic logarithm is calculated as −log R/R_0_.

## 3. Results and Discussion

### 3.1. UV Spectroscopy

There are numerous references for the APIs in solution [19,20,26,27] in the UV range, but for solid API drug samples suitable references were not found. For this reason, first the liquid solutions of the APIs were measured and then compared to the results found in the literature. In a second step, samples in the solid phase, i.e., the pure API reference samples and the commercial painkillers, were investigated.

#### 3.1.1. APIs in Liquid Phase, Transmission Spectroscopy

**Figure 4:**
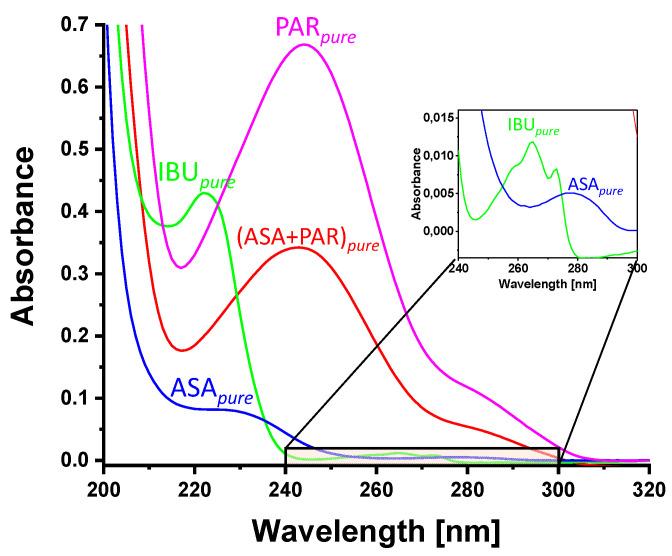
UV absorbance spectra of APIs ibuprofen (IBU), acetylsalicylic acid (ASA), paracetamol (PAR) and a mixture of acetylsalicylic acid and paracetamol (ASA+PAR) in liquid phase.

The absorbance of IBU*_pure_*, ASA*_pure_*, PAR*_pure_* as well as a mixture of ASA*_pure_* with PAR*_pure_* in liquid solution were analyzed in the UV range. Figure 4 shows their absorption spectra in the UV region (200–320 nm). The smaller features of IBU*_pure_* and ASA*_pure_* in the range of 240–300 nm are shown in the inset in Figure 4. All samples show a strongly increasing absorbance below 310 nm. IBU*_pure_* presents one prominent maximum at 223 nm and three weaker maxima located at approximately 258 (sh), 265 and 273 nm. ASA*_pure_* exhibits a broad maximum at approximately 228 nm and a further, more pronounced but less intense maximum at around 277 nm. PAR*_pure_* shows a distinct band with a maximum at 244 nm and a weak shoulder at 284 nm. The mixture of ASA*_pure_* and PAR*_pure_* presents a band maximum at 240 nm and a shoulder at 282 nm. These findings are listed in Table 2 [19,20,27]. The determined band positions are consistent with those reported by Saeed et al. (2016) and Lawson et al. (2017) [20,27].

#### 3.1.2. API and Painkiller Tablets, Total Hemispherical Reflectance Spectroscopy

**Figure 5:**
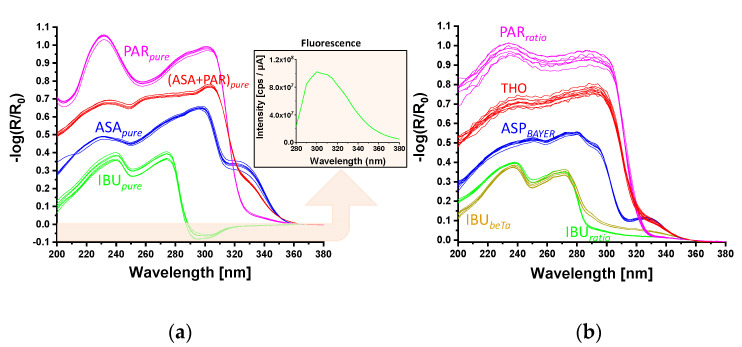
UV total hemispherical reflectance spectra of drug samples in the solid phase in the wavelength range 200–380 nm. (**a**) API drugs IBU*_pure_*, ASA*_pure_*, PAR*_pure_* and a mixture of ASA*_pure_* with PAR*_pure_*. Upper right: Fluorescence emission of IBU sample with excitation at 270 nm. (**b**) Painkiller tablets IBU*_ratio_*, IBU*_beTa_*, ASP*_ratio_*, PAR*_ratio_* and THO.

Two sample sets of tablets were used to study the total hemispherical reflectance in the solid phase (see Figure 1 and Table 1). The first set consisted of pure APIs: IBU*_pure_*, ASA*_pure_* and PAR*_pure_* and a mixture of ARA*_pure_* and PAR*_pure_*. The second set consisted of commercial painkiller tablets. Three samples from each API were prepared and analyzed. Figure 5 shows the preprocessed reflectance spectra of solid samples in the UV region (200–380 nm). Spectra were recorded from each side of the samples (Figure 5a).

The most striking feature is the negative reflectance of IBU*_pure_* in the wavelength range of 288–340 nm, which is due to fluorescence emission (inset in Figure 5a). All spectra show several contributions, which are listed in Table 2.

The painkiller tablets were measured at different depth levels, i.e., one layer of approximately 500 µm was removed from the samples after each measurement; the resulting spectra are shown in Figure 5b. The similarity of the spectra at all depth levels inside the tablets indicates an almost regular distribution of ingredients. Although IBU*_ratio_* and IBU*_beta_* were manufactured from different companies, they show similar spectral characteristics. The most prominent contributions are also listed in Table 2.

The comparison of the spectra from the APIs and commercial painkiller tablets indicates that the overall spectral characterizations are comparable. Nevertheless, several deviations are observed. The spectral features are more pronounced in the API samples, also the negative absorbance observed in the IBU sample is absent in the commercial painkillers. The reason for these differences is mainly that the commercial tablets do not have 100% API content. For example, the IBU*_ratio_* tablets contains additionally pregelatinized corn starch, hypromellose, croscarmellose sodium, stearic acid, highly dispersed silicon dioxide, macrogol 8000, titanium dioxide. Some of these substances show some absorption in selected spectral range i.e., titanium dioxide shows a pronounce absorbance [28]. Since the exact percentage of the composition is not known, a final statement on the influence of these substances on the spectra cannot be made.

### 3.2. UV Hyperspectral Imaging

#### 3.2.1. API Tablets, Hyperspectral Imaging

**Figure 6:**
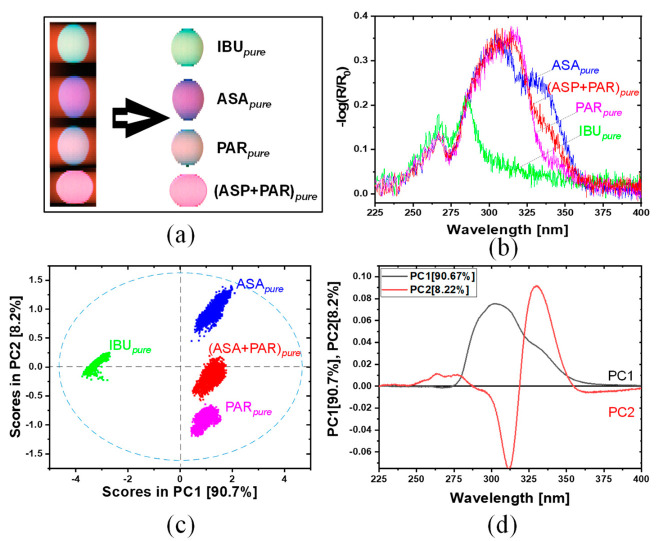
(**a**) Raw hyperspectral image for all API drug samples before and after subtracting the background. (**b**) Spectrum recorded for a single pixel of each of pure API samples in the UV range 225–400 nm. (**c**,**d**) Scores and corresponding loadings plot.

Figure 6 shows the results of UV hyperspectral imaging in the range from 225 to 400 nm. Figure 6a shows the raw image before (left) and after subtraction of the background (right). Figure 6b shows a spectrum of an arbitrary but representative pixel for each API sample. The most dominant contribution for IBU*_pure_* is observed around 275 nm and for PAR*_pure_* at around 305 nm. For ASA*_pure_*, two strong contributions at 300 and 330 nm are observed. The mixture of (ASA+PAR)*_pure_* shows—as expected—a combination of the spectral properties of ASA*_pure_* and PAR*_pure_*. In the range 255–270 nm, all API preparations show a small peak in their reflectance at around 265 nm. Towards lower wavelengths, the spectra show no additional features.

In the next step, a PCA model with cross validation (venetian blinds, 10 splits, 1 sample per split) was calculated for the spectra of all preparations. The first two PCs explain 98.9% of the total variance. Figure 6c shows the scores plot of the PC1 and PC2. The scores plot shows that PC1 and PC2 are sufficient to separate all samples clearly from one another. PC1 yields a clear separation of IBU*_pure_* from the other APIs, whereas the remaining APIs are separated with PC2. The mixture (ASA+PAR)*_pure_* is found almost in the middle between ASA*_pure_* and PAR*_pure_*.

The loadings plot for PC1 and PC2 is given in Figure 6d. The loading of PC1 is dominated by an overall positive contribution in the range between 280 and 350 nm, PC2 shows one more narrow negative contribution at 311 nm and one positive at 330 nm.

The comparison between the shape of spectra shown in Figure 5a or Figure 6b shows similarities as well as some clear deviations. The shape of the spectra of all APIs is quite well reproduced in the range above 275 nm. Most striking in this range is an intensity deviation of the different spectral bands, i.e., for the ASA*_pure_* the shoulder at 330 nm is much more pronounced in the hyperspectral imaging spectra. The same is valid also for the PAR*_pure_* sample. In the range below 275 nm clear deviations are observed. The shape of the spectra shown in Figure 6b is characterized by a continuously decreasing intensity, whereas the spectra of the APIs in Figure 5a show clear variations in their shape in this range (see also below).

**Figure 7:**
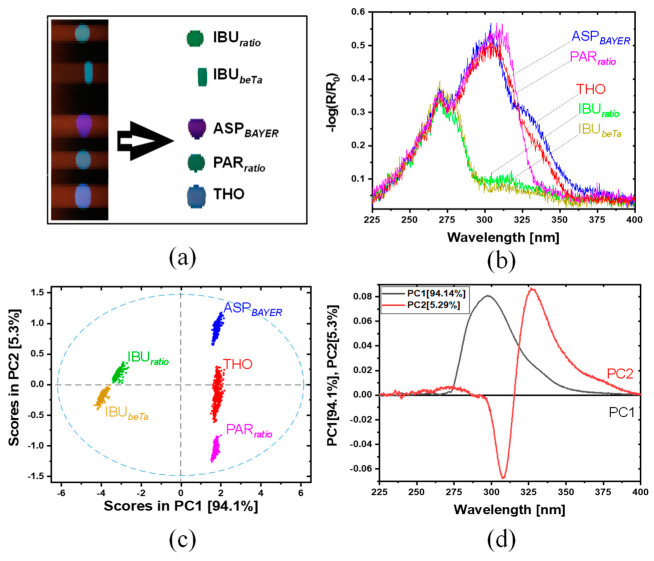
(**a**) Raw hyperspectral image for all commercial painkiller tablets before and after subtracting the background. (**b**) Spectrum recorded for a single pixel of each painkiller tablet in the UV range 200–400 nm. (**c**,**d**) Scores and corresponding loadings plot.

Figure 5 presents UV spectra with a good signal-to-noise-ratio recorded with a research grade UV desktop spectrometer. Here, the measured area for one spectrum consisted of 12 × 5 mm. Figure 6 and Figure 7 show UV spectra with a less good signal-to-noise-ratio recorded with the UV hyperspectral imager. These spectra result from one single pixel of the detector, representing a much smaller area of the tablet which considered of 13 × 13 µm. A further reason for the low signal-to-noise-ratio is weak irradiation intensity in the HSI setup by an XBO lamp.

#### 3.2.2. Commercial Painkiller Tablets, Hyperspectral Imaging

Figure 7 shows results from the hyperspectral imaging of the second sample set, consisting of commercial painkiller tablets, in the range from 225 to 400 nm. Figure 7a shows the raw image before (left) and after (right) subtraction of the background. Figure 7b shows a spectrum of an arbitrary but representative pixel for each painkiller samples. For IBU*_ratio_* and IBU*_beta_*, the most dominant contribution is observed around 270 nm, and a further contribution with much lower intensity is observed at around 315 and 333 nm. The spectra of IBU*_ratio_* and IBU*_beTa_* are quite similar; it seems that the contributions from the further added chemical ingredients are spectroscopically comparable. The spectrum for ASP*_BAYER_* is dominated by a broad intensity distribution at around 304 nm. Here, two contributions of different intensity are specifiable, a more intense with maximum at 304 nm and a weaker one at 333 nm. For PAR*_ratio_*, only one strong contribution with maximum at 310 nm is observed; whereas THO shows two contribution of different intensity, a more intense with maximum at 304 nm and a weaker one at 333 nm. In the range 225–275 nm, all painkiller samples show a minor peak in their reflectance at around 270 nm. Towards lower wavelength, the spectra show no additional features.

The shape of the spectra of the commercial painkiller match those of the APIs (Figure 6b) quite well. Slight deviations are most likely due to additional ingredients in the commercial samples.

Figure 7d shows the loadings plot for PC1 and PC2. PCA model was calculated by cross validation (venetian blinds, 10 splits, 1 sample per split). The loading of PC1 is dominated by an overall positive contribution in the range between 275 and 350 nm, whereas PC2 shows one more narrow negative contribution at 308 nm and one positive at 327 nm. The distribution of the clusters in the scores plot in Figure 7c shows a comparable variability with the scores plot in Figure 6c. Only for the THO sample an increased spreading is observed along PC2. In general, such type of variability in the shape of the cluster can arise for several reasons; a change in the sample’s properties on the scale of the resolution actually achieved, or changes due to shape effects of the samples or positioning within the hyperspectral imaging setup. A general reason for deviations between the hyperspectral imaging and UV-Vis spectroscopy (see Figure 5 vs. Figure 6b or Figure 7b) are the different geometries used for illumination and detection in the setups. In the UV spectrometer the light is collected in an almost perfectly reflecting integrating sphere, while in case of the UV hyperspectral imaging, a tunnel made of PTFE is used for illumination and collecting as shown in Figure 2a. As a consequence, a clear differentiation between specular and diffuse reflection is not possible in the hyperspectral imaging setup, therefore, a mixture of both contributions will be detected here.

Comparing the hyperspectral imaging spectra in Figure 6b or Figure 7b with the spectra given in Figure 5 it is clear that the hyperspectral imaging data provide almost no useful spectroscopic information in the region < 275 nm. The low performance in this range is due to the efficiency of detector and the illumination in the hyperspectral imaging setup. A further consequence of this is that contributions at higher wavelengths appear more dominant as they actually are. The tendency of increasing sensitivity exists for the entire wavelength range. This is also why the shoulders observed in the spectra of ASA/ASP and IBU at >25 nm (in both sample sets) appear much more enhanced compared to the spectra in Figure 5. As a consequence, the actual hyperspectral imaging setup yields valuable results for all samples, but reliable spectroscopic information is only accessible in the range above 275 nm, and there, attention must be paid to the relative intensities. Despite the spectroscopy weaknesses, the combination of UV hyperspectral imaging and chemometric modeling enables a complete separation of all samples in both sample sets. The loadings plots (Figure 6c or Figure 7c) indicate that a differentiation of all samples is possible considering only a few spectral channels, so that rapid classification is easily possible.

In order to validate the pure API PCA model (see Figure 6), the scores of PC1 and PC2 were used to calculate a quadratic discriminant analysis (QDA). The confusion matrix resulted from this model is listed in Table 3. A confusion matrix describes the performance of the classification model based on QDA. An overall accuracy for the pure API tablets of 99.8% is reached, which means the model can correctly classify approximately all spectra of the pure API tablets. The highlighted diagonal describes how many spectra were predicted by the model as true. Only 19 spectra of (ASA+PAR)*_pure_* were predicted as PAR*_pure_* and two spectra of PAR*_pure_* as (ASA+PAR)*_pure_*. This is because (ASA+PAR)*_pure_* contains both API components (ASA*_pure_*, PAR*_pure_*).

This QDA model was used to classify all spectra of the painkiller tablets. Even 99.8% of the spectra were predicted correctly (see Table 4). This means approximately all painkiller tablets were predicted correctly in true API classes. Only two spectra of ASP*_BAYER_* were assigned as (ASA+PAR)*_pure_*. This is because (ASA+PAR)*_pure_* contains both API components (ASA*_pure_*, PAR*_pure_*).

The UV region is often preferred in process control and quality assurance, but hyperspectral imaging in this region is rarely reported. The aim of this study was to develop a simple UV hyperspectral imaging setup capable of distinguishing between different drug samples as an example for a possible industrial application. With the prototype, a painkiller table can be measured at 4 s. This speed is adequate for scientific purposes, but too low for industrial applications. The limiting factor towards a setup for a production environment is the intensity of the illumination and the quantum yield of the pushbroom imager. With an appropriate light source and imager then this setup is capable for in-line data acquisition, process control, in-line classification/sorting, and thus real-time release testing.

## 4. Conclusions

UV hyperspectral imaging was used to characterize active pharmaceutical ingredients in tablets. Two sample sets were analyzed; sample set one consisted of tablets with 100% API content and sample set two consisted of commercially available painkiller tablets. Reference measurements were performed on the pure APIs in liquid solutions and in solid phase using a commercial UV spectrometer.

Hyperspectral imaging in combination with PCA is a promising approach for the detection and differentiation of all drug samples studied. The PCA model was able to separate all drug types with the first two principle components. The advantage of the home-built setup is a high spatial/spectral resolution and a data acquisition speed completely sufficient for scientific studies. Based on the design and the data shown, a setup fulfilling the requirements of a real industrial process can be easily realized.

## Figures and Tables

**Table 1:** Types of drug samples.

Samples	Descriptions	Abbreviation	Manufacturer	CAS Number
**Ibuprofen**	Ibuprofen, >98%, API	IBU*_pure_*	Caesar & Loretz GmbH, Hilden, Germany	15687-27-1
**Acetylsalicylic acid**	Acetylsalicylic acid, 99%, API	ASA*_pure_*	Acros organics, New Jersey, US	50-78-2
**Paracetamol**	Paracetamol, 99%, API	PAR*_pure_*	Hebei Jiheng (Group) Pharmaceutical Co., Ltd.	103-90-2
**Ibuprofen**	Ibuprofen (400 mg)	IBU*_ratio_*	Ratiopharm GmbH, Ulm, Germany	-
**Ibuprofen**	Ibuprofen (400 mg)	IBU*_beTa_*	Betapharm, Arzneimittel GmbH, Augsburg, Germany	-
**Aspirin**	Acetylsalicylic acid (500 mg)	ASP*_BAYER_*	Bayer Vital GmbH, Leverkusen, Germany	-
**Paracetamol**	Paracetamol (500 mg)	PAR*_ratio_*	Ratiopharm GmbH, Ulm, Germany	-
**Thomapyrin**	Thomapyrin (250 mg acetylsalicylic acid/ paracetamol, 50 mg coffin)	THO	Sanofi-Aventis GmbH, Frankfurt, Germany	-

**Table 2:** UV band maxima positions of liquid and solid phase samples [11,19,20].

Drug Type	API Liquid Phase	API Solid Phase	Painkiller Tablets Solid Phase
**IBU**	223 nm 258 nm (sh) 265 nm 273 nm	240 nm 275 nm	238 nm (IBU*_ratio_*, IBU*_beTa_*) 275 nm (IBU*_ratio_*, IBU*_beTa_*)
**ASA/ASP**	228 nm 277 nm	230 nm 277–310 nm 328 nm (sh)	228 nm (ASP*_BAYER_*) 280 nm (ASP*_BAYER_*) 294 nm (ASP*_BAYER_*) 329 nm (ASP*_BAYER_*)
**PAR**	244 nm 284 nm	232 nm 305 nm	233 nm (PAR*_ratio_*) 300 nm (PAR*_ratio_*)
**THO**	-	-	238 nm 331 nm
**ASA+PAR (mixture)**	240 nm 282 nm	235 nm 277–332 nm	-

**Table 3:** The confusion matrix of the pure API spectra.

Predicted					
**Actual**	API Samples	IBU*_pure_*	ASA *_pure_*	PAR*_pure_*	(ASA+PAR)*_pure_*
	IBU*_pure_*	2365	0	0	0
	ASA *_pure_*	0	2428	0	0
	PAR*_pure_*	0	0	2574	2
	(ASA+PAR)*_pure_*	0	0	19	2586

**Table 4:** Classification of the painkiller tablets based on the pure API model.

Predicted					
**Actual**	Samples	IBU*_pure_*	ASP *_pure_*	PAR*_pure_*	(ASA+PAR)*_pure_*
	IBU*_ratio_*/IBU*_beTa_*	469	0	0	0
	ASP *_BAYER_*	0	283	0	2
	PAR*_ratio_*	0	0	209	0
	THO	0	0	0	394

## Data Availability

The data presented in this study are available on request from the corresponding author. The data are not publicly available due to privacy restrictions.
